# Survival After Risk-Reducing Mastectomy in Patients With Pathogenic Variants in *BRCA1* and *BRCA2*: A Network Meta-Analysis

**DOI:** 10.1001/jamanetworkopen.2026.41231

**Published:** 2026-10-30

**Authors:** Isabel Beshar, David Viveros-Carreño, Nuria Agusti, Karla Barajas, Kate J. Krause, Maria Angeles Lopez-Olivo, Maria D. Iniesta, Banu Arun, Helen M. Johnson, Alexander Melamed, Roni Nitecki Wilke, J. Alejandro Rauh-Hain

**Affiliations:** 1 Department of Gynecologic Oncology and Reproductive Medicine, The University of Texas MD Anderson Cancer Center, Houston; 2 Grupo de Investigación Salud de la Mujer Sanitas, Department of Gynecologic Oncology, Clínica Colsanitas S.A., Clínica Universitaria Colombia, Grupo de Investigación GIGA, Centro de Tratamiento e Investigación Sobre Cáncer Luis Carlos Sarmiento Angulo (CTIC), Bogotá, Colombia; 3 Research Medical Library, The University of Texas MD Anderson Cancer Center, Houston; 4 Department of Health Services Research, Division of Cancer Prevention and Population Sciences, The University of Texas MD Anderson Cancer Center, Houston; 5 Department of Breast Medical Oncology, The University of Texas MD Anderson Cancer Center, Houston; 6 Department of Breast Surgical Oncology, The University of Texas MD Anderson Cancer Center, Houston; 7 Department of Gynecologic Oncology, Vincent Department of Obstetrics and Gynecology, Mass General Brigham, Boston, Massachusetts

## Abstract

**Importance:**

The independent survival benefit of risk-reducing mastectomy (RRM), separate from risk-reducing salpingo-oophorectomy (RRSO), remains uncertain among patients with pathogenic variants (PVs) in *BRCA1* and *BRCA2* genes.

**Objective:**

To evaluate the association of RRM, magnetic resonance imaging (MRI)-based screening, and mammography alone with all-cause and breast cancer–specific mortality using RRSO-adjusted estimates.

**Data Sources:**

MEDLINE, Embase, and Web of Science were searched from January 1, 1990, to May 28, 2026. Search structures, subject headings, and keywords were tailored to each database.

**Study Selection:**

Inclusion criteria were English-language prospective and retrospective observational studies of adult female patients with PV or likely PV in *BRCA1* and *BRCA2* who underwent RRM or breast cancer screening (MRI-based with or without mammography, or mammography alone), with RRSO excluded by design or adjusted for in multivariable analysis. Studies including females with prior breast or ovarian cancer were excluded.

**Data Extraction and Synthesis:**

Study reporting followed Preferred Reporting Items for Systematic Reviews and Meta-Analyses 2020 network meta-analysis extension. Two authors independently performed study selection, data extraction, and bias assessment using a risk-of-bias assessment tool (ROBINS-I V2) and a grading approach (Grading of Recommendations Assessment, Development, and Evaluation [GRADE]). Frequentist random-effects network meta-analysis was performed with surface under the cumulative ranking curve (SUCRA) estimation. Sensitivity analysis was conducted for the *BRCA1* cohort.

**Main Outcomes and Measures:**

Primary outcome was all-cause mortality. Secondary outcome was breast cancer–specific mortality. All outcomes were analyzed and reported as hazard ratios (HRs) with corresponding 95% CIs.

**Results:**

Five observational studies with 7937 female patients (median [range] age, 35.9 [unreported] years to 43.4 [32.0-55.7] years) met the inclusion criteria. RRM was associated with lower all-cause mortality compared with MRI-based screening (HR, 0.37; 95% CI, 0.22-0.63) and mammography alone (HR, 0.16; 95% CI, 0.08-0.32). RRM was also associated with lower breast cancer–specific mortality compared with mammography alone (HR, 0.07; 95% CI, 0.01-0.44) but did not have significantly lower mortality than MRI-based screening (HR, 0.26; 95% CI, 0.05-1.35). In *BRCA1*-only analyses, RRM was associated with lower breast cancer–specific mortality compared with MRI-based screening (HR, 0.13; 95% CI, 0.03-0.66) and mammography alone (HR, 0.03; 95% CI, 0.00-0.15). SUCRA rankings indicated RRM had the highest likelihood of association with the lowest all-cause mortality (SUCRA value, 99.9%). Risk of bias was critical for 4 studies, and certainty of evidence was rated as very low for all 5 studies.

**Conclusions and Relevance:**

In this systematic review and network meta-analysis of RRSO-adjusted analyses, RRM was associated with lower all-cause mortality compared with breast cancer screening among females with PVs in *BRCA1* and *BRCA2*, with the greatest magnitude of association for breast cancer–specific mortality observed in *BRCA1* PV carriers. These findings provide less confounded survival estimates to support individualized shared decision-making for RRM.

## Key Points

**Question:**

What is the survival benefit of risk-reducing mastectomy (RRM), independent of risk-reducing salpingo-oophorectomy (RRSO), among patients with pathogenic variants (PVs) in *BRCA1* and *BRCA2* genes?

**Findings:**

In this network meta-analysis of 5 observational studies with 7937 female patients, RRM was associated with a significant decrease in all-cause mortality compared with breast cancer screening with magnetic resonance imaging or mammography.

**Meaning:**

The findings of this study suggest that RRM may provide survival benefit beyond that attributable to RRSO among patients with PVs in *BRCA1* and *BRCA2* genes and should remain a central component of individualized risk-reduction counseling.

## Teaser

This meta-analysis evaluates all-cause and breast cancer–specific deaths after risk-reducing mastectomy among *BRCA1* and *BRCA2* pathogenic variant carriers in prospective and retrospective observational studies.

## Introduction

Adult females with pathogenic variants (PVs) in *BRCA1* and *BRCA2* face a cumulative lifetime breast cancer risk exceeding 70% and may be offered risk-reducing mastectomy (RRM) or recommended intensive screening to mitigate these risks.^1^ Although RRM consistently reduces breast cancer incidence, its association with long-term survival remains uncertain.^1,2^ A 2018 Cochrane review found inconsistent evidence of improvement in overall and disease-specific survival among females who underwent RRM, identifying risk-reducing salpingo-oophorectomy (RRSO) as a major confounder given its established survival benefit in this population.^2^ RRSO has been associated with substantial reductions in ovarian cancer incidence, ovarian cancer–specific mortality, and all-cause mortality among *BRCA1* and *BRCA2* PV carriers and may additionally affect breast cancer risk through reduction in circulating estrogen levels.^3,4,5^ As a result, the independent survival benefit associated with RRM remains difficult to isolate in observational studies in which many patients undergo both interventions.

Despite surgical advances, RRM remains a major procedure, with reported complication rates as high as 30% to 40%.^2,6^ The morbidity and permanency of this procedure, with its documented effects on quality of life and body image, paired with uncertainty regarding its survival benefit, create a complex risk-benefit tradeoff for patients and clinicians.^7,8,9^ Current guidelines recommend RRM as an option without explicitly endorsing it, reflecting this uncertainty.^1^ For patients who select screening, lifelong breast imaging is recommended, including annual magnetic resonance imaging (MRI) beginning at age 25 years and mammography starting at age 30 years until age 75 years.^1^

Quantifying the long-term survival of RRM is essential, as decisions to pursue RRM are currently made in the absence of definitive comparative survival data, but utilization is increasing.^10^ Prior studies that did not account for RRSO risk overestimating the independent association between RRM and survival.^2^ To address this gap and rank comparative associations among patients with PVs in *BRCA1* and *BRCA2*, we conducted a systematic review and network meta-analysis. Our aim was to evaluate the association of RRM, MRI-based screening, and mammography alone with all-cause and breast cancer–specific mortality using RRSO-adjusted estimates.

## Methods

### Protocol and Registration

This systematic review and network meta-analysis followed the 2020 Preferred Reporting Items for Systematic Reviews and Meta-Analyses (PRISMA) Extension for Network Meta-Analysis^11,12^ reporting guideline. Our protocol was registered in the International Prospective Register of Systematic Reviews (PROSPERO; 420251141021).

### Eligibility Criteria

Inclusion criteria were English-language prospective and retrospective observational studies on adult females with PV or likely PV in *BRCA1* and *BRCA2* genes who underwent RRM or screening and with summary estimates of all-cause and breast cancer–specific mortality reported using hazard ratios (HRs). We defined screening as MRI-based (MRI with or without mammography) or mammography alone to account for heterogeneity in both clinical adherence and age-based screening strategies among patients with PVs in *BRCA1* and *BRCA2*; current guidelines support annual breast MRI at age 25 years, followed by alternating MRI and mammography starting at age 30 years, such that included cohorts may reasonably include patients undergoing distinct imaging modalities across the follow-up period. We included studies that specifically excluded RRSO in study design or adjusted for the procedure in multivariable analysis given its established independent survival benefit in *BRCA* PV carriers.^4^ We excluded studies with patients reporting a history of breast or ovarian cancer to avoid skewing cancer-specific mortality outcomes, studies with no comparison groups, and studies with insufficient data.

### Information Sources and Search Strategy

We searched Ovid MEDLINE, Ovid Embase, and Web of Science. Databases were searched from January 1, 1990, to May 28, 2026. Search structures, subject headings, and keywords were tailored to each database by a medical research librarian specializing in systematic reviews (K.J.K.) in consultation with coauthors. Searches were restricted to English language articles including human participants and excluded case reports, conference materials, and ongoing clinical trials. The full search strings for all databases and other resources can be found in eMethods 1 in Supplement 1, in accordance with the PRISMA-S guideline.^12^

### Study Selection

After the initial search, a systematic review software (Covidence; Veritas Health Innovation Ltd) was used to screen the citations. Four of us (I.B., N.A., M.D.I., and K.B.) independently screened the titles and abstracts of the articles to identify potentially relevant studies. Disagreements on article inclusion were resolved by consensus and by seeking the opinion of another reviewer (D.V.-C.) when consensus could not be reached. Studies that passed the title and abstract review were retrieved for a full-text review. Two of us (I.B. and D.V.-C.) independently screened the remaining full-text articles for inclusion, and disagreements were resolved by seeking the opinion of another author (N.A.). When multiple publications reported results from the same patient consortium, we included only the most recent report (eMethods 2 in Supplement 1).

### Data Collection

Two of us (I.B. and D.V.-C.) independently extracted data using a data form created with the systematic review software, with our disagreements resolved by discussion with another author (N.A.). We considered studies to have sufficient data if at least 1 of the listed primary outcomes and selected time points were reported. Data extraction included publication data (author names and publication year), study location and design, sample size, inclusion and exclusion criteria, participants’ features (age, PV, and follow-up time), and details of interventions (type, approach, frequency, and comparator intervention).

### Risk of Bias Within Studies

Two of us (I.B. and D.V.-C.) independently assessed the risks of bias for each study and resolved disagreements through consultation with a third author (N.A.). We used a risk-of-bias assessment tool (ROBINS-I V2; Cochrane) to evaluate studies across the following domains: confounding, classification of intervention, selection into the study, missing data, measurement of the outcome, and selection of the reported result. Each potential source of bias was graded as low, moderate, serious, or critical; an overall grade was determined based on the highest risk attributable to any individual domain.

### Summary Measures and Geometry of the Network

The primary outcome was all-cause mortality, defined as death from any cause. The secondary outcome was breast cancer–specific mortality. All outcomes were analyzed and reported as HRs with corresponding 95% CIs. HRs were extracted from study summary estimates and analyzed on the log scale, with SEs derived from the published 95% CIs. We prioritized age-adjusted HRs reported by the studies. In development of the network geometry, we predefined 3 intervention nodes: mammography alone, MRI-based screening, and RRM, with mammography alone as the prespecified reference intervention.

### Assessment of Inconsistency and Risk of Bias Across Studies

We planned a priori to assess local inconsistency within the network using node splitting, which evaluates direct and indirect evidence for each treatment comparison. If network geometry or sample size precluded statistical tests, we planned to assess key study-level variables for consistency across nodes. We considered age at intervention or observation as a key potential effect modifier in our assessment of transitivity given its association with both RRM and mortality, and we extracted available age measures from each included study and compared age distributions across intervention nodes. Formal age-stratified network meta-analysis or meta-regression was not performed because included studies did not consistently report age-stratified HRs. Other key study-level variables that could vary population attributes and modify survival estimates were included, such as location, calendar year, distribution of PVs, and follow-up time. To assess bias within studies, we planned for funnel plots with associated Egger tests if more than 10 studies reported on the same outcome.

### Certainty of Evidence

Certainty of evidence was assessed using the Grading of Recommendations Assessment, Development, and Evaluation approach.^11^ We rated the certainty for each comparison and outcome as high, moderate, low, or very low, based on consideration of risk of bias, inconsistency, indirectness, publication bias, intransitivity, incoherence, and imprecision.

### Statistical Analysis

We conducted a frequentist random-effects model network meta-analysis. Separate estimates for *BRCA1* and *BRCA2* PV carriers without an overall combined estimate were pooled within each applicable study’s data using a fixed-effect inverse-variance approach on the log HR scale before inclusion in the network meta-analysis (eMethods 3 in Supplement 1). As a post hoc sensitivity analysis to assess the robustness of our findings, we investigated for unmeasured confounding in the primary comparison for all-cause mortality using the E*-*value, which captures the minimum magnitude of association that an unmeasured confounder would need to have with both RRM and overall survival, conditional on measured covariates, to fully explain the observed association.^13^ HRs were treated as approximating risk ratios under a rare-outcome assumption.

To rank the interventions, we estimated rank probabilities and calculated surface under the cumulative ranking curve (SUCRA) values. SUCRA values range from 0% to 100%, with higher values indicating a greater likelihood of association with superior treatment performance.^14,15^ When data were available, we conducted a subgroup analysis for breast cancer–specific mortality in *BRCA1* PV carriers, as this subgroup comprised the majority of included patients and accounted for most breast cancer mortality events across the studies. We also conducted a sensitivity analysis removing single-center studies to assess whether study setting affected the association between RRM and mortality. All analyses were conducted in R using the netmeta and meta packages (R Project for Statistical Computing).

## Results

Five observational studies with 7937 female patients (median [range] age, 35.9 [unreported] years to 43.4 [32.0-55.7] years) met inclusion criteria.^16,17,18,19,20^ We originally retrieved 835 unique articles for review (Figure 1). Of these, 14 studies underwent full-text review and 9 studies were excluded, most commonly due to the absence of all-cause or breast cancer–specific mortality data (eTable 1 in Supplement 1).

**Figure 1.**
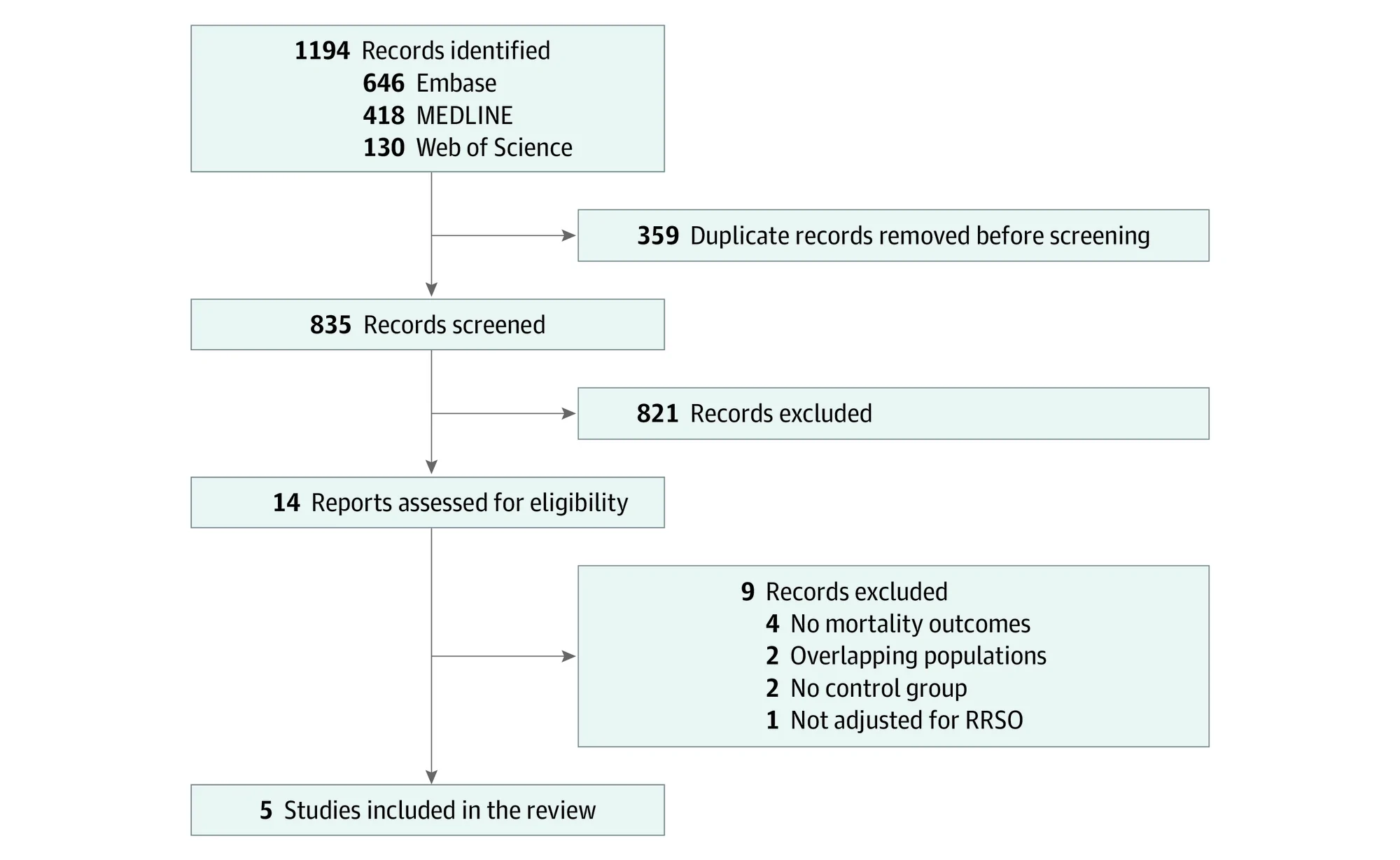
PRISMA Flow Diagram The flow diagram outlines the identification, screening, eligibility assessment, and inclusion of studies. RRSO indicates risk-reducing salpingo-oophorectomy.

### Network Geometry and Study Characteristics

The geometry of our analysis is presented in Figure 2. Included studies were published between 2013 and 2024, representing data collected between 1980 and 2022. There was 1 prospective single-center cohort study,^16^ 1 prospective multicenter case-control study,^18^ 1 prospective multicenter cohort study,^19^ and 2 retrospective cohort studies^17,20^ (Table 1). Reported follow-up time spanned a median (range) of 3.5 (3.1-4.3) years to 13.3 (unreported) years. All studies included both patients with PVs in *BRCA1* and *BRCA2*, with variants in *BRCA1* making up the majority in 3 of the 5 cohorts.^17,18,19^ Record et al^20^ did not include RRSO by study design, and all other studies accounted for RRSO using multivariable regression adjustment.^16,17,18,19^

**Figure 2.**
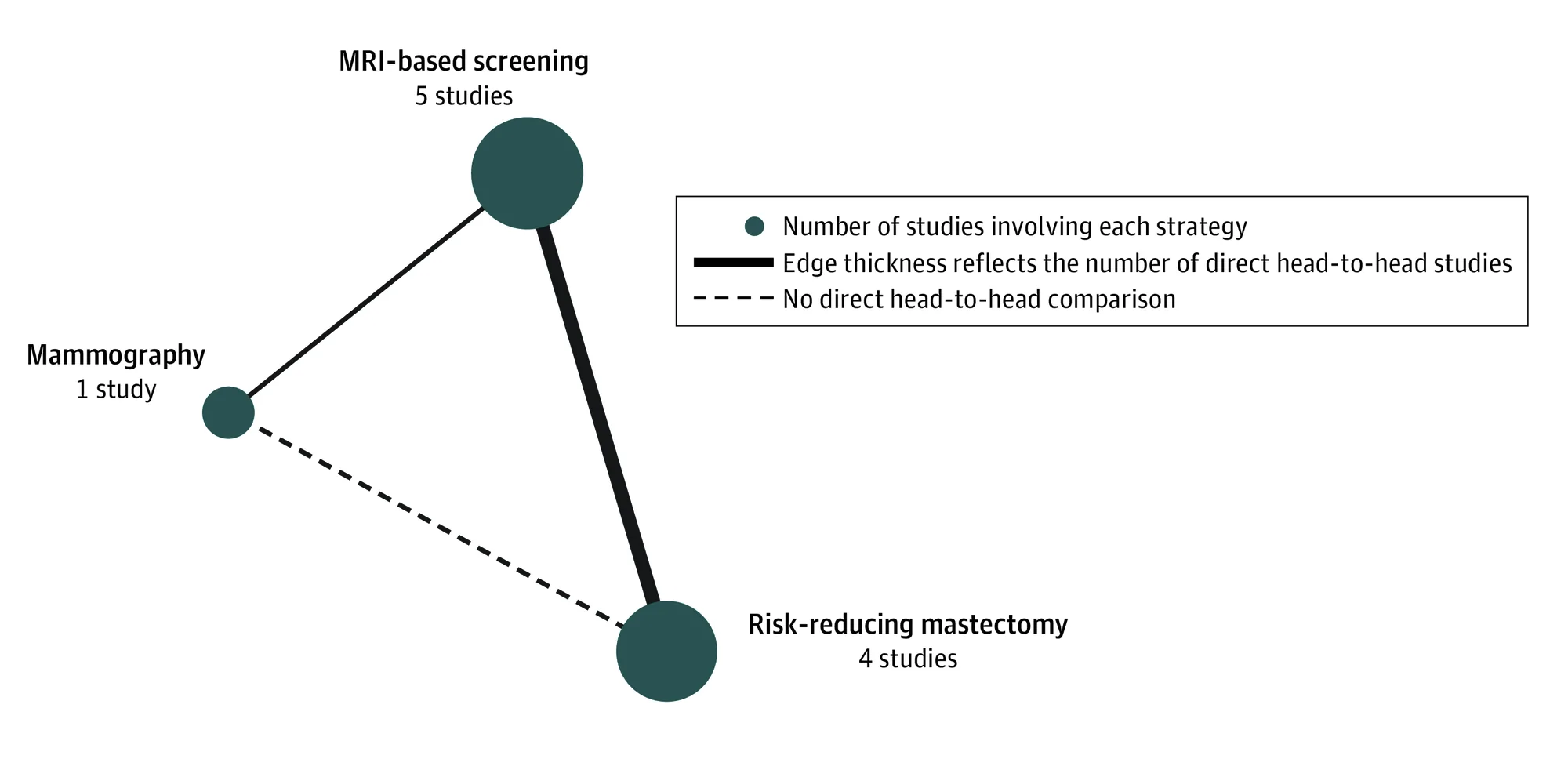
Geometry Structure of Network Meta-Analysis MRI indicates magnetic resonance imaging.

**Table 1.** Study Characteristics

Source; study location	Study type	Sample size, No.	Treatment group, No.	Control group	Age, median (range), y	Follow-up time, median (range), y	Pathogenic variant distribution, No.
Ingham et al,^16^ 2013; United Kingdom	Prospective, single-center cohort study	691	RRM: 126; control: 219	MRI-based screening	35.9 [NR]	13.3 [NR]	*BRCA1*: 346; *BRCA2*: 345
Heemskerk-Gerritsen et al,^17^ 2019; Netherlands	Retrospective, multicenter cohort study	2857	RRM: 1128; control: 1729	MRI-based screening	38.0 (34.0-42.0)^a^	8.5 (7.2-8.5)^a^	*BRCA1*: 1712; *BRCA2*: 1145
Metcalfe et al,^18^ 2024; international	Prospective, multicenter, case-control	1654	RRM: 827; control: 827	MRI-based screening	Mean (range): 42.3 (20.0-71.0)	Mean (range): 6.3 (1.0-23.9)	*BRCA1*: 568; *BRCA2*: 259
Record et al,^20^ 2024; US	Retrospective, single-center cohort study	247^b^	RRM: 57; control: 190	MRI-based screening	43.4 (32.0- 55.7)	3.5 (3.1-4.3)	*BRCA1*: 134^b^; BRCA2: 172^b^
Lubinski et al,^19^ 2024; international	Prospective, multicenter cohort study	2488	MRI: 1756; control: 732	Mammography	41.2 (30.0-69.0)	5.3 (0.1-21.0)	*BRCA1*: 2004; *BRCA2*: 484

Abbreviations: MRI, magnetic resonance imaging; NR, not reported; RRM, risk-reducing mastectomy.

^a^ Medians were calculated from combining group medians in the study.

^b^ Study included additional male patients (n = 59) for a total cohort size of 306, but males were excluded from the survival analysis. Separate numbers for males and females are not provided for *BRCA1* and *BRCA2*; thus, the full cohort of pathogenic variants is provided here.

### Risk of Bias Within Studies

The summary of risk-of-bias assessment is provided in the eFigure in Supplement 1. One study—Metcalfe et al^18^—was found to be at moderate risk of bias, and the remaining articles were assessed to be at critical risk of bias.^16,17,19,20^ The primary factor associated with critical risk across studies was self-selection bias into surgical intervention. There was also concern for immortal time bias (ie, time from diagnosis of PV to RRM or screening), although some studies (Lubinski et al^19^ and Heemskerk-Gerritsen et al^17^) attempted to mitigate this association by using time-dependent analyses.^17,19^

### All-Cause Mortality

RRM was associated with lower all-cause mortality than MRI-based screening (HR, 0.37; 95% CI, 0.22-0.63) and mammography alone (HR, 0.16; 95% CI, 0.08-0.32) (Figure 3; eTable 2 in Supplement 1). SUCRA plots suggested a hierarchy among interventions, with RRM ranked as having the highest likelihood of association with the lowest all-cause mortality (SUCRA value, 99.9%) followed by MRI-based screening (SUCRA value, 49.9%), while mammography alone was ranked as having the lowest likelihood (SUCRA value, <0.0%) (Table 2). Estimates were unchanged in the sensitivity analysis that removed single-center studies (Record et al^20^ and Ingham et al^16^). RRM was associated with lower all-cause mortality than MRI-based screening (HR, 0.42; 95% CI, 0.22-0.77) and mammography alone (HR, 0.18; 95% CI, 0.08-0.39). The E-value for RRM and all-cause mortality, compared with MRI-based screening, was 4.85 for the point estimate and 2.66 for the 95% CI limit, indicating that an unmeasured confounder would need risk ratio associations of at least 4.85 with both RRM and mortality to explain the association and that associations of 2.66 could shift the interval to the null. For RRM vs mammography alone, E-values were 11.98 for the point estimate and 5.70 for the 95% CI limit, indicating that a more robust unmeasured confounder would be required.

**Figure 3.**
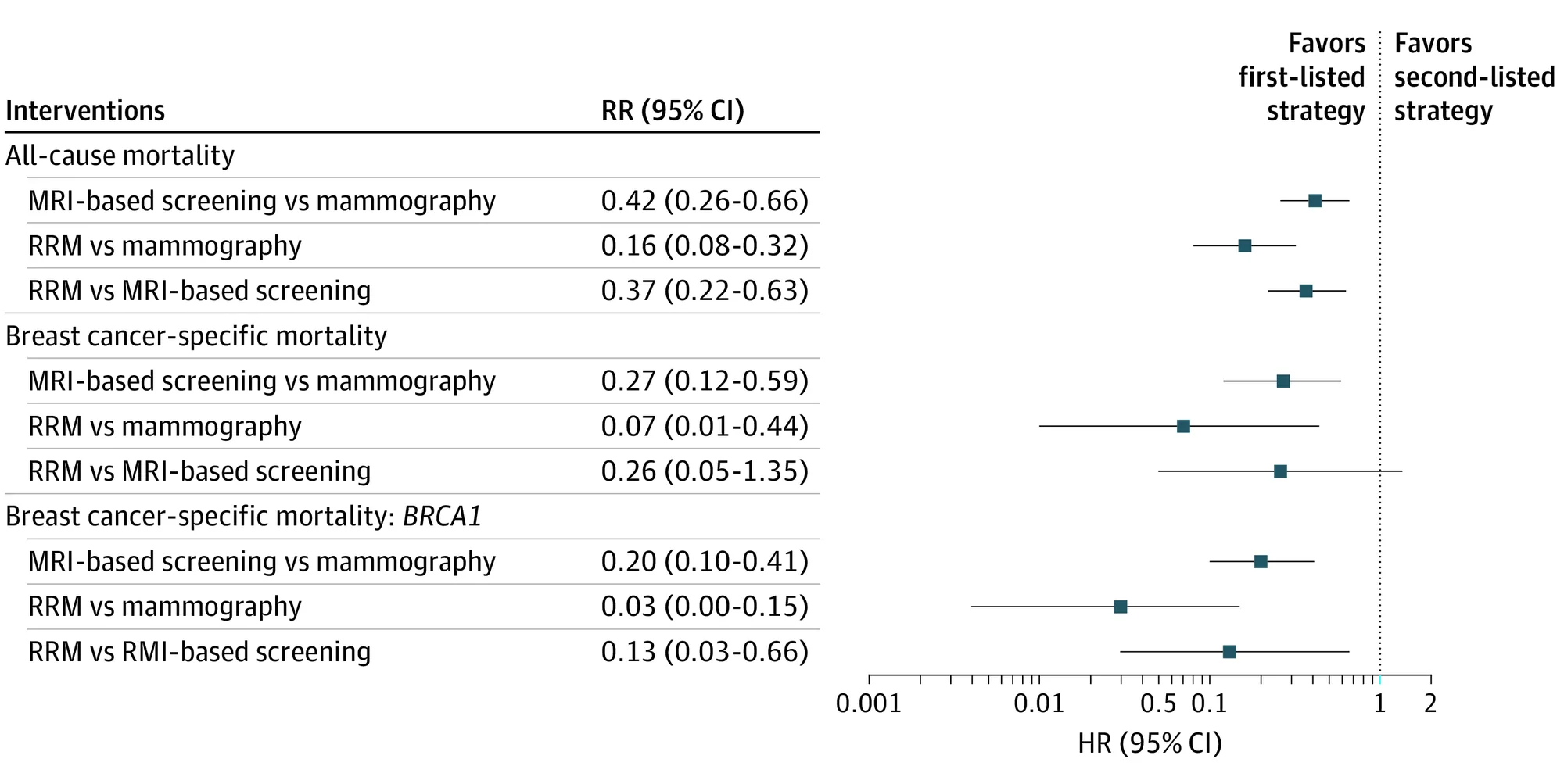
Forest Plots for All Network Comparisons Using Random-Effects Network Meta-Analysis Data are presented as pooled hazard ratios (HRs) with 95% CIs. Error bars represent 95% CIs. MRI indicates magnetic resonance imaging, and RRM, risk-reducing mastectomy.

**Table 2.** SUCRA Rankings for Estimated Network Meta-Analysis Associations^a^

Association	Probability, %			SUCRA value,%
	Best	Modest	Worst	
**All-cause mortality**				
Mammography alone	0.0000	0.0131	99.9869	<0.0
MRI-based screening	0.0109	99.9760	0.0131	49.9
RRM	99.9891	0.0109	0.0000	99.9
**Breast cancer–specific mortality**				
Mammography alone	0.0041	0.2684	99.7275	0.1
MRI-based screening	5.4495	94.4982	0.0523	52.6
RRM	94.5464	5.2334	0.2202	97.1

Abbreviations: MRI, magnetic resonance imaging; RRM, risk-reducing mastectomy; SUCRA, surface under the cumulative ranking curve.

^a^ Totals may not sum to 100 because of rounding.

### Breast Cancer–Specific Mortality

RRM was associated with lower breast cancer–specific mortality than mammography alone (HR, 0.07; 95% CI, 0.01-0.44) but not significantly lower mortality than MRI-based screening (HR, 0.26; 95% CI, 0.05-1.35) (Figure 3). In the sensitivity analyses that included only patients with PVs in *BRCA1*, RRM was associated with lower breast cancer–specific mortality in both MRI-based screening (HR, 0.13; 95% CI, 0.03-0.66) and mammography alone groups (HR, 0.03; 95% CI, 0.00-0.15). SUCRA values for breast-cancer specific survival were 97.1% for RRM, 52.6% for MRI-based screening, and 0.1% for mammography alone.

### Inconsistency, Risk of Bias, and Certainty of Evidence

Because our network geometry was not a closed loop, we could not perform statistical tests for network transitivity and instead compared key study-level variables for inconsistency, including location, calendar period, design, sample size, screening exposure, participant age, follow-up period, distribution of PVs, adjustment of covariates, oophorectomy handling, and treatment details (eTable 3 in Supplement 1). No complete information was provided for specific genetic testing, MRI access, mammography quality, surgical techniques, and systemic therapy use, among others.

Risk of publication bias could not be assessed statistically with funnel plots because fewer than 10 studies were included in our analysis. Certainty of evidence was very low for all comparisons in the network due to the observational design of all contributing studies. No study used prospective randomized clinical trial methods (eTable 4 in Supplement 1).

## Discussion

In this systematic review and network meta-analysis, RRM was associated with lower all-cause mortality compared with MRI-based screening among *BRCA1* and *BRCA2* PV carriers, even when accounting for RRSO as a key survival-related confounder. Among patients with PVs in *BRCA1* specifically, RRM was also associated with lower breast cancer–specific mortality compared with screening modalities. To our knowledge, this study is the first meta-analysis to prioritize RRSO-adjusted estimates when evaluating the association between RRM and survival among *BRCA1* and *BRCA2* PV carriers, addressing a major source of confounding that has limited interpretation of prior studies. Because a randomized clinical trial of RRM is unlikely to be feasible, well-designed observational studies with rigorous confounder adjustment represent the highest attainable level of evidence for this question. Our findings suggest RRM may provide an independent survival benefit among patients with PVs in *BRCA1* and *BRCA2*, particularly among *BRCA1* PV carriers, and should remain a central component of individualized risk-reduction counseling.

Uptake of RRM has increased substantially over the past 2 decades, coinciding with expanded access to genetic testing and growing public awareness, yet evidence supporting a survival benefit remains mixed.^21^ The disconnect between uptake and evidence is clinically relevant because RRM carries perioperative risks, physical consequences, and long-term psychosocial effects, including changes in body image and sexual function, making accurate survival estimates essential for informed shared decision-making.^9^ A recent meta-analysis of 6 observational studies, including 6135 patients with PVs in *BRCA1* and *BRCA2*, reported an 81% to 86% reduction in breast cancer–related mortality but did not systematically adjust for RRSO or specify screening completed by the control group, which may inflate estimates of benefit and limit attribution of survival specifically to RRM.^22^ Conversely, Gandhi et al^23^ found no statistically significant survival benefit with RRM despite marked reduction in breast cancer incidence across 1205 patients in the UK with PVs in *BRCA1* and *BRCA2*; however, groups were substantially imbalanced by RRSO status (62% in the RRM group vs 34% in the screening group), and no adjustment in the survival estimates was performed. These studies highlight the challenges in isolating the independent survival benefit of RRM and underscore the need for analyses that rigorously account for confounders and varying screening strategies.

Increasing effectiveness of MRI-based screening with earlier-stage detection may attenuate the absolute benefit associated with RRM compared with historical cohorts. Nevertheless, we observed substantial overall survival advantage for RRM even compared with MRI-based screening protocols, including in cohorts with more than 1 decade of follow-up time and screening exposure, although the magnitude of benefit appeared smaller than that observed with mammography alone. Ultimately, our network analysis enabled indirect comparison of RRM with mammography alone, demonstrating the relative inferiority of mammography alone in this high-risk population and underscoring the limited effectiveness of screening without adjunct MRI or surgical risk reduction.

In this study, RRM was not associated with a statistically significant reduction in breast cancer–specific mortality compared with MRI-based screening in the overall population with *BRCA1* and *BRCA2* PV, although a *BRCA1*-restricted sensitivity analysis suggested a potential benefit. No formal interaction test between variant subgroups was performed given the absence of *BRCA2*-only estimates in most of the included studies, as no patients opting for RRM died during the study periods. As a result, these findings should be considered with caution. *BRCA1-*associated breast cancers are more aggressive, predominately triple-negative tumors that have fewer targeted options, which may partially explain the greater relative reduction in breast cancer–specific mortality observed in the *BRCA1-*restricted group.^24^ In contrast, *BRCA2-*associated breast cancers are more commonly hormone-receptor positive, with increasingly more effective endocrine and other targeted therapies available; a potential incremental survival benefit of RRM may therefore be more difficult to detect given the smaller number of females with PVs in *BRCA2* across included studies, lower event rates, and a more favorable baseline prognosis. Larger studies with *BRCA2*-specific estimates are needed before conclusions can be drawn about variant-differential benefit.

### Limitations

This study has limitations. The findings must be interpreted within important methodological constraints. All studies comparing RRM with screening were observational, and most of them were rated at critical risk of bias due to self-selection of patients opting for surgery or screening. Individuals who select RRM and those who pursue screening may differ in ways that independently affect survival, including comorbidity burden, health behaviors, and access to high-quality care. In Record et al,^20^ cardiovascular and pulmonary comorbidities were independently associated with a lower likelihood of undergoing RRM, providing empirical support for this selection dynamic. Age is an important potential modifier in this setting, with expected survival benefit of RRM anticipated to vary by age at diagnosis, age at RRM, duration of prior screening exposure, and competing mortality risk. We evaluated age descriptively as part of the transitivity assessment and study-specific HRs were age-adjusted, but available aggregate data did not permit formal testing of age-by-intervention interaction. Although several studies used multivariable adjustment and E-values suggested that substantial unmeasured confounding would be required to fully explain the observed associations, residual confounding cannot be definitively excluded and would be expected to favor the RRM group, potentially leading to the overestimation of the association of RRM with survival. Immortal time bias arising from the window between PV diagnosis and RRM is an additional concern for which some, but not all, studies attempted to account for and, if present, would favor the surgical group. The network lacked a closed loop, and key comparisons, including RRM vs mammography alone, were based on indirect evidence, requiring the assumption of transitivity across studies. Finally, gray literature was not included in this analysis, and publication bias cannot be definitively assessed.

## Conclusions

In RRSO-adjusted analyses, RRM was associated with lower all-cause mortality compared with breast cancer screening among female patients with PVs in *BRCA1* and *BRCA2*, with the association for breast cancer–specific mortality being of greatest magnitude among *BRCA1* PV carriers. By prioritizing estimates that account for RRSO, these findings provide more precise data to support individualized shared decision-making about the expected survival benefit of RRM.

## Notes

Supplement 1. **eMethods 1.** Information Sources and Search Strategies **eMethods 2.** Managing Records **eMethods 3.** Statistical Analysis **eFigure.** Risk of Bias in Non-Randomized Studies of Interventions (ROBINS-I-V2) **eTable 1.** List of Studies Excluded From Analysis and Rationale **eTable 2.** League Table for Mortality Estimates Using Random Effects Network Meta-Analysis **eTable 3.** Study-Level Variables Relevant to Transitivity Across Network Comparisons **eTable 4.** Certainty of Evidence Using the Recommendations Assessment, Development, and Evaluation (GRADE) Approach
eReferences

Supplement 2.
Data Sharing Statement
