# Supplementary material for: Survival After Risk-Reducing Mastectomy in Patients With Pathogenic Variants in BRCA1 and BRCA2: A Network Meta-Analysis
Source: JAMA Netw Open. 2026 Oct 30;9(10):e2641231. doi: 10.1001/jamanetworkopen.2026.41231 (PMC13723018; doi:10.1001/jamanetworkopen.2026.41231)
Supplement: Supplementary file 1 [file jamanetwopen-e2641231-s001.pdf]

Supplemental Online Content

Beshar I, Viveros-Carreño D, Agusti N, et al. Survival after risk-reducing mastectomy in patients with pathogenic variants in *BRCA1* and *BRCA2*: a network meta-analysis. *JAMA Network Open*. 2026;9(10):e2641231. doi:10.1001/jamanetworkopen.2026.41231

eMethods 1. Information Sources and Search Strategies

eMethods 2. Managing Records

eMethods 3. Statistical Analysis

eFigure. Risk of Bias in Non-Randomized Studies of Interventions (ROBINS-I-V2)

eTable 1. List of Studies Excluded From Analysis and Rationale

eTable 2. League Table for Mortality Estimates Using Random Effects Network Meta-Analysis

eTable 3. Study-Level Variables Relevant to Transitivity Across Network Comparisons

eTable 4. Certainty of Evidence Using the Recommendations Assessment, Development, and Evaluation (GRADE) Approach

eReferences

This supplemental material has been provided by the authors to give readers additional information about their work.

© 2026 Beshar I et al. *JAMA Network Open*.

eMethods 1: Information Sources and Search Strategies

Databases searched included Ovid MEDLINE, Ovid EMBASE, and Web of Science. No study registries were searched. Search structures, subject headings, and keywords were tailored to each database by a medical research librarian (KJK) specializing in systematic reviews in consultation with co-authors. Searches were restricted to human and English language articles and excluded case reports, conference materials, editorials, letters, surveys, and biographies. Databases were first searched from January 1, 1990 to September 22, 2025; a second search with the same methodology was then performed to include articles published until May 28, 2026. The electronic search strategy was performed in accordance with PRISMA-S. Contact with study authors was sought for one study (Record et al.). See search string provided at the end of this document.

© 2026 Beshar I et al. *JAMA Network Open*.

eMethods 2: Managing Records

1,194 records were identified, including 418 from Medline, 646 from Embase, and 130 from Web of Science. Deduplication of 359 records was performed manually in Endnote. After the initial search, Covidence software was used to screen the citations. Two of the principal investigators independently screened the titles and abstracts of the articles to identify potentially relevant studies, with disagreements resolved by consensus and by seeking the opinion of a third reviewer. This resulted in 14 reports. Studies that passed the title/abstract review were retrieved for full-text review. The two investigators then independently screened the remaining full-text articles, with disagreements resolved by consensus and by seeking the opinion of a third reviewer. 9 studies were excluded.

© 2026 Beshar I et al. *JAMA Network Open*.

eMethods 3: Statistical Analysis

Network meta-analyses were performed within a frequentist framework using hazard ratios (HRs) as the treatment-effect measure. HRs were analyzed on the log scale, and standard errors were derived from the published 95% confidence intervals. Three intervention nodes were prespecified: mammography alone, MRI-based screening with or without mammography, and risk-reducing mastectomy. Mammography alone was used as the reference intervention.

Random-effects network meta-analysis was used for all-cause mortality in the overall *BRCA1/2* population and for breast cancer-specific mortality in the *BRCA1*-restricted analysis. A fixed-effect model was used for breast cancer-specific mortality in the overall *BRCA1/2* population because the network contained too few replicated study-level contrasts to estimate between-study heterogeneity reliably. When separate *BRCA1* and *BRCA2* estimates were reported without an overall estimate, log HRs were combined within study using fixed-effect inverse-variance weighting before inclusion in the network meta-analysis.

For sensitivity analyses requiring a risk-ratio scale, HRs were treated as approximating risk ratios under a rare-outcome assumption. Treatment rankings were estimated using rank probabilities and summarized with Surface Under the Cumulative Ranking curve values. Ranking was oriented so that lower mortality corresponded to a more favorable rank. SUCRA values range from 0 to 1, with higher values indicating a greater probability of being among the interventions associated with lower mortality. Rank probabilities and SUCRA values were estimated using the same network model specified for each outcome. The values themselves are intended to incorporate uncertainty from the underlying treatment estimate.[1] Because rankings are model-

© 2026 Beshar I et al. *JAMA Network Open*.

based summaries and may be unstable in sparse networks, they were interpreted descriptively and in conjunction with relative effect estimates and certainty of evidence.

© 2026 Beshar I et al. *JAMA Network Open*.

eFigure: Risk of Bias in Non-Randomized Studies – of Interventions (ROBINS-I-V2)

Figure includes the consensus on the authors' rankings of the risk of bias for each study. Dark red circles, red circles, yellow circles, and green circles indicate critical risk, high risk, moderate risk, and low risk, respectively. D1: Confounding; D2: Classification of the intervention; D3: Selection into the study; D4: Missing data, D5: Measurement of outcome; D6: Selection of reported result

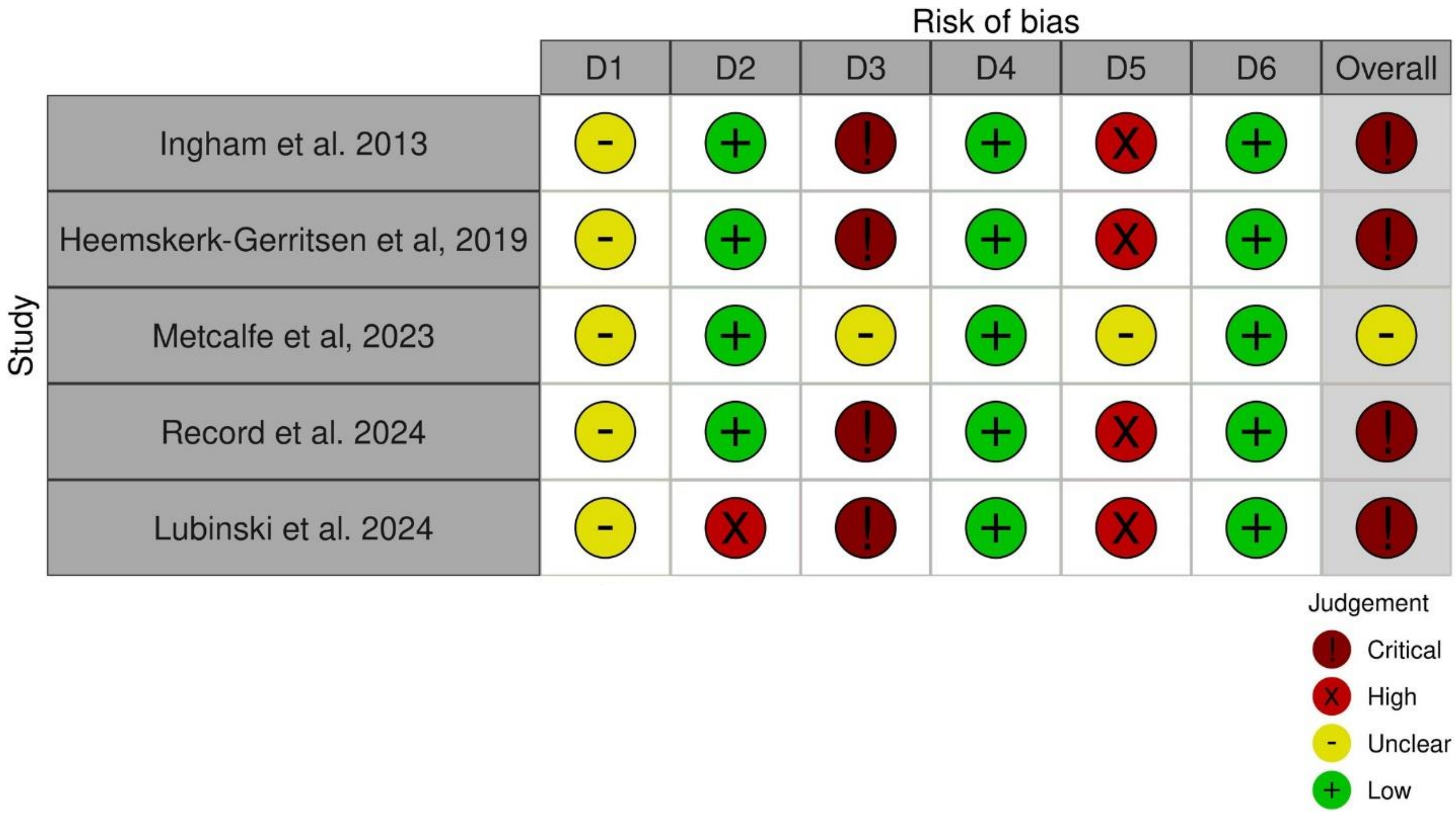

© 2026 Beshar I et al. *JAMA Network Open*.

eTable 1: List of studies excluded from analysis and rationale

Source	Study Type	Intervention and Comparator	Rationale for Exclusion
Evans et al. 2014[2]	Prospective cohort	MRI-based screening vs mammography	Overlapping patient population
Heemskerk-Gerritsen et al. 2013[3]	Prospective cohort	RRM vs. screening	Overlapping patient population<br>Comparator intervention not specified
Valero et al. 2020[4]	Prospective cohort	RRM	No control group
Warner et al. 2020[5]	Prospective cohort	MRI	No control group
Domchek et al. 2010[6]	Prospective cohort	RRM vs. screening	No mortality outcomes
Passaperuma et al. 2012[7]	Prospective cohort	MRI-based screening vs mammography	No mortality outcomes (no hazard ratios provided)
Evans et al. 2009[8]	Prospective cohort	RRM vs screening<br>RRSO vs screening	No mortality outcomes (for RRM sub-group)
Willert et al. 2025[9]	Retrospective case control	RRM vs no surgery<br>RRSO vs no surgery	Comparator intervention not specified<br>Not adjusted for RRSO-status
Gandhi et al. 2026[10]	Prospective cohort	RRM vs MRI-based screening	No mortality outcome (no hazard ratio provided)<br>Not adjusted for RRSO-status

Abbreviations: RRM, risk reducing mastectomy; MRI, magnetic resonance imaging; RRSO, risk-reducing salpingo-oophorectomy

© 2026 Beshar I et al. *JAMA Network Open*.

eTable 2: League table for mortality estimates using random effects network meta-analysis. Data are presented as pooled hazard ratios (95% confidence intervals)

	**Mammography**	**MRI-based**	**RRM**
All-cause Mortality			
**Mammography**	—	2.38 (1.52–3.85)	6.37 (3.16–12.81)
**MRI-based**	**0.42 (0.26–0.66)**	—	2.67 (1.59–4.50)
**RRM**	**0.16 (0.08–0.32)**	**0.37 (0.22–0.63)**	—
Breast Cancer-Specific Mortality			
**Mammography**	-	3.70 (1.68–8.14)	14.25 (2.29–88.49)
**MRI-based**	**0.27 (0.12–0.59)**	-	3.85 (0.74-19.99)
**RRM**	**0.07 (0.01–0.44)**	0.26 (0.05–1.35)	-
Breast Cancer Specific Mortality for BRCA1			
**Mammography**	-	5.00 (2.41–10.37)	38.77 (6.47–232.15)
**MRI-based**	**0.20 (0.10–0.41)**	-	7.75 (1.51–39.75)
**RRM**	**0.03 (0.00–0.15)**	**0.13 (0.03–0.66)**	-

Abbreviations: MRI, magnetic resonance imaging, RRM, risk reducing mastectomy.

© 2026 Beshar I et al. *JAMA Network Open*.

eTable 3: Study-level variables relevant to transitivity across network comparisons

Network Comparison	Study, country, ascertainment period	Design/Setting	Sample size and analytic groups	Intervention and comparator	Age and follow up	Adjustment	Risk-reducing oophorectomy	Incident breast cancers, chemotherapy data
**RRM vs MRI-based**								
**Ingham et al. 2013**[11]	United Kingdom; Europe; years not provided	Prospective single-center cohort	691 total; RRSO 457; RRM 126; Control 219	RRM versus screening program (defined as MRI and mammography)	34.4 years for *BRCA-1*; 37.4 years for *BRCA-2*; follow up 13.3 (no ranges)	Age, PV	BRRM and RRSO as separate time-dependent covariates in a multivariable Cox proportional hazards model.<br><br>*BRCA1* (56 RRSO only, 29 RRM only, 42 both surgeries); *BRCA2* (52 RRSO only, 29 RRM only, 26 both surgeries)	34 breast cancers (*BRCA1*); 38 breast cancers (*BRCA2*); no chemotherapy data provided
**Heemskerk-Gerritsen et al. 2019**[12]	Netherlands, Europe; 1995-2016	Retrospective multi-center national cohort (eight academic centers plus the Netherlands Cancer Institute)	2,857 total; RRM 722 *BRCA1* and 406 *BRCA2;* Control 990 *BRCA1* and 739 *BRCA2*	RRM vs screening (defined as annual MRI age 25-60 since 1998, annual mammography age 30-60, mammography after age 60, and annual clinical	Age at start of observation: *BRCA1* RRM 34 (29-41) and screening 38 (30-47) and *BRCA2* RRM 36 (29-43) and screenin	Age (year of birth, age at observation, age at DNA test), year of DNA test, and RRSO	RRSO was included in the propensity score as a time-dependent variable. RRSO was more common in RRM groups: *BRCA1* 557/722 (77%) vs	276 breast cancers (*BRCA1*); 144 breast cancers (*BRCA2*) Chemotherapy *BRCA1* 170/273 (62%); *BRCA2* 56/142 (39%)

© 2026 Beshar I et al. *JAMA Network Open*.

				breast exam).	g 42 (33-51); follow up 10.3 years (no range)		569/990 (57%); *BRCA2* 293/406 (72%) vs 441/739 (60%).	
**Metcalfe et al. 2024**[13]	International, Europe, North America, Western Asia; cases 2006.3 (1994-2018) and controls 2006.6 (1992-2019)	Prospective multi-center international registry analysis using pseudo-randomized design (58 centers in 9 countries including Canada, United States, Poland, Norway, Austria, Israel, Italy, Netherlands, Spain)	1,654 initially cancer-free women, 827 matched pairs. RRM 827; control 827. Eligible matches from pool of 4625	RRM vs no RRM; with sub-analysis for those who underwent breast MRI MRI exposure common but not universal (673/827, 84.0% in controls and 639/827, 74.9% in cases)	Mean age at index date controls 42.6 (21-71), RRM 42.0 (20-70). Mean follow-up from index date 6.3 y (range 0.1-23.9); controls 6.6 (0.1-23.9), RRM 6.1 (0.0-23.5).	Matched on age, year of birth, and country of residence	RRSO adjusted as a time-dependent variable. Bilateral oophorectomy: controls 499/827 (60.3%), RRM 626/827 (75.7%).	120 breast cancers; 100/827 (12.1%) of controls and 20/827 (2.4%) of cases Chemotherapy 50/79 (63.3%) among controls; did not provide for cases
**Record et al. 2024**[14]	United States; Duke Hereditary Cancer Clinic; no start date until April 2020	Retrospective single-center cohort	306 total carriers, including 247 female patients; total RRM 58 and controls 248<br><br>Female analytic comparison: RRM 57/247 (23.1%) vs no RRM 190/247	All patients recommended to undergo annual breast MRI/ and annual mammography; patient-level data not provided in paper or by authors upon request.	All patients median age at genetic testing 43.4 y (IQR 32.0-55.7); no RRM 45.7 (32.3-57.5), RRM 39.3 (31.1-46.3). Median follow-up 41.6 months (95% CI	PV, marital status, systemic illness comorbidities, benign breast disease	No patients with RRSO included	Female patients: 2 malignancies detected at RRM and 1 breast cancer after RRM. No chemotherapy data reported.

© 2026 Beshar I et al. *JAMA Network Open*.

					36.8-51.0)			
**MRI-based vs mammography**								
**Lubinski et al. 2024**[15]	International; North America-Caribbean, Europe, Western Asia; 1995-2015	Prospective multi-center cohort from 59 centers in 11 countries (United States, Canada, Poland, Norway, Israel, Italy, Austria, Netherlands, France, Spain, Bahamas)	2,488 women with *BRCA1*/2 (2004 *BRCA1*; 484 *BRCA2*); MRI 1,756; no MRI 732.	MRI program vs no MRI. No MRI group, 636/732 (86.7%) reported having had at least 1 mammogram; in MRI group 1544/1756 (87.9%) had at least 1 mammogram	Age at start of follow-up: no MRI 42.2 (30-69), MRI 43.2 (30-69). Overall mean follow-up from baseline 9.2 y (range 0.1-24.5).	Age at study entry, PV, country of residency, RRSO	RRSO adjusted as time-dependent covariate; RRSO no MRI 333/732 (45.5%), MRI 1,155/1,756 (65.8%).	344 total breast cancers; MRI 241 vs no MRI 103. Invasive BC: MRI 205 vs no MRI 79; DCIS: MRI 33 vs no MRI 17. Chemotherapy noted as a variable but not reported.

Abbreviations**:** RRM = risk-reducing mastectomy; RRSO = risk-reducing salpingo-oophorectomy; BC = breast cancer; DCIS = ductal carcinoma in situ. Age, follow-up, and chemotherapy variables are not harmonized across studies and should be reported as study-level descriptors, not pooled node-level estimates.

© 2026 Beshar I et al. *JAMA Network Open*.

eTable 4: Certainty of evidence using the Recommendations Assessment, Development, and Evaluation (GRADE) Approach

Certainty Assessment							Effect			
**Number of studies**	**Study design**	**Risk of bias**	**Inconsistency**	**Indirectness**	**Imprecision**	**Other considerations**	**Relative effect, HR (95% CI)**	**Absolute effect, HR (95% CI)**		
**Question:** Risk-reducing mastectomy compared to MRI-based screening for patients with *BRCA* pathogenic variant										
4	Non-randomized studies	Very serious	Not serious	Serious	Not serious	Strong association	0.37 (0.22–0.63)	0 fewer per 1,000[a]	⊕◯◯◯ Very low	Critical
**Question:** MRI-based screening compared to mammography alone for patients with *BRCA* pathogenic variant										
1	Non-randomized studies	Very serious	Not serious	Serious	Not serious	Strong association	0.42 (0.26–0.66)	0 fewer per 1,000[a]	⊕◯◯◯ Very low	Critical
**Question:** Risk-reducing mastectomy compared to mammography alone for patients with *BRCA* pathogenic variant										
5	Non-randomized studies	Very serious	Not serious	Serious	Not serious	None	0.16 (0.08–0.32)	0 fewer per 1,000[a]	⊕◯◯◯ Very low	Critical

Abbreviation: HR, hazard ratio.

[a] The absolute effect in these studies was between 0 and 1 fewer per 1,000 patients.

© 2026 Beshar I et al. *JAMA Network Open*.

SEARCH STRING

OVID EMBASE

1. "Hereditary Breast and Ovarian Cancer Syndrome"/
2. *BRCA1* protein/
3. *BRCA2* protein/
4. (*BRCA1* or *BRCA2*).ti,ab.
5. (BRCA*1 or BRCA*2).ti,ab.
6. (hereditary adj3 (breast or ovarian) adj3 cancer*).ti,ab.
7. (familial adj3 (breast or ovarian) adj3 cancer*).ti,ab.
8. HBOC.ti,ab.
9. (genetic* predispos* adj3 (breast or ovarian) adj3 cancer*).ti,ab.
10. OR/1-9 [HBOC]
11. Prophylactic Surgical Procedure/
12. ((preemptive or "pre-emptive" or prophylactic* or prevent* or "risk-reduc*" or "risk reduc*") ADJ2 (surger* or surgical or procedure*)).ti,ab.
13. Prophylactic Mastectomy/
14. ((bilateral or double or contralateral) adj3 mastectom*).ti,ab.
15. ((preemptive or pre-emptive or prophylactic* or preventiv* or prevent* or risk-reduc* or "risk reduc*") ADJ2 (mastectom*)).ti,ab.
16. Risk-Reducing Bilateral Salpingo-Oophorectomy/
17. ("risk-reduc*" ADJ2 "salpingo-oophorectom*").ti,ab.

© 2026 Beshar I et al. *JAMA Network Open*.

18. ((preemptive or pre-emptive or prophylactic* or preventiv* or prevent* or risk-reduc* or "risk reduc*") ADJ2 ("salpingo-oophorectom*")).ti,ab.
19. Salpingectomy/
20. Exp Salpingooophorectomy/
21. (oophorectom* or ovariectom* or salpingectom* or "salpingo-oophorectom*").ti,ab.
22. OR/11-21 [preventative surgeries]
23. Exp Mammography/
24. (mammograph* or mammogram* or "breast magnetic resonance" or "breast computed tomograph*" or echomammogra* or xeromammography).ti,ab.
25. OR/23-24 [mammograms]
26. Exp Oral Contraceptive Agent/
27. ("oral contraceptive*" or "oral contraception" or "birth control pill*" or "contraceptive agent*" or "contraceptive pill*").ti,ab.
28. (Chlormadinone or Cyproterone or Deposiston or Desogestrel or Drospirenone or Estradiol or "Ethinyl Estradiol" or Etynodiol or Levonorgestrel or Lynestrenol or Medroxyprogesterone or Mestranol or Norethisterone or Norethindrone or Norgestrel or Meloxifene or Segesterone or Tanaproget).Ti,Ab.
29. Anastrozole/
30. (Anastrozole or Arimidex or Femathina or Zolitrat).ti,ab.
31. Exp Aromatase inhibitor/
32. (aromasin or "aromatase inhibitor*" or aminoglutethimide or exemestane or letrozole).ti,ab.
33. Raloxifene/
34. (Evista or Loxifen or Ortruma or Ostiral or Raloxifene).ti,ab.

© 2026 Beshar I et al. *JAMA Network Open*.

35. Selective Estrogen Receptor Modulator /
36. ("estrogen receptor modulat*" or "oestrogen receptor modulat*").ti,ab.
37. Tamoxifen/
38. Tamoxifen Citrate/
39. (Tamoxifen or Nolvadex or Tamofen* or Soltamox).ti,ab.
40. Exp Intrauterine Contraceptive Device/
41. ("intrauterine contraceptive device*" or "intrauterine device*" or IUD or "I.U.D." or levonorgestrel).ti,ab.
42. OR/26-41[all drug terms]
43. OR/22,25,42 [prophylactic surgeries, mammograms, contraception]
44. AND/10,43 [HBOC and interventions]
45. Mortality/
46. Cancer Mortality/
47. All Cause Mortality/
48. Mortality Rate/
49. Surgical Mortality/
50. (cancer adj2 (mortalit* or death or fatalit*)).ti,ab.
51. ((death or fatality or mortality) ADJ rate).ti,ab.
52. "all-cause mortalit*".ti,ab.
53. ("overall survival" or "cancer-specific survival").ti,ab.
54. "survival outcome*".ti,ab.
55. (survival or mortality or death*).ti.
56. (("ovar* cancer" or "ovar* carcinoma" or "ovar* metasta*") ADJ2 (death* or mortality)).ti,ab.

© 2026 Beshar I et al. *JAMA Network Open*.

57. (("breast cancer" or "breast carcinoma" or "breast metasta*") ADJ2 (death* or mortality)).ti,ab.
58. (("endometrial cancer" or "endometrial carcinoma" or "endometrial metasta*") ADJ2 (death* or mortality)).ti,ab.
59. (("colo* cancer" or "colo* carcinoma" or "colo* metasta*") ADJ2 (death* or mortality)).ti,ab.
60. (("fallopian tube* cancer*" or "fallopian tube* carcinoma" or "fallopian tube* metasta*") ADJ2 (death* or mortality)).ti,ab.
61. Proportional Hazards Model/
62. Survival Analysis/
63. "survival analys*".ti,ab.
64. ("relative risk*" or "absolute risk*").ti,ab,kw.
65. (hazard adj2 ratio*).ti,ab,kw.
66. ("HR" ADJ2 (confidence or CI)).ti,ab,kw.
67. ("cox regression" or "cox model*" or "cox multivariate" or "cox survival").ti,ab,kw.
68. ("proportional hazard*" or "hazard ratio*").ti,ab,kw.
69. OR/45-68 [mortality reduction terms]
70. AND/44, 69 [HBOC and interventions and mortality]
71. (exp Animal/ OR exp Animal Model/ or exp Animal Experiment/ or Nonhuman/ or "In Vitro Study"/) NOT exp Human/
72. (amphibian* or animal? or bird or birds or bovine or canine or cat or cats or cattle or cow or cows or dog or dogs or ferret* or fish or frog* or "guinea pig*" or hamster* or horse* or "in vitro" or lambs or llama* or marmoset* or mice or monkey* or mouse or murine or pig or pigs or piglets or porcine or primate or primates or rabbit* or rat or rats or sheep or swine or trout* or veterinar* or zebra*).ti.

© 2026 Beshar I et al. *JAMA Network Open*.

73. OR/71,72 [animal studies]

74. 70 NOT 73 [results without animal studies]

75. Abstract.pt. or Overall/

76. Abstract Report/

77. Book/ or Book.pt.

78. Exp Biography/ or biograph*.ti.

79. (book and review*).ti.

80. Case Report/ or Case Reports/

81. Chapter/ or Chapter.pt.

82. Conference/ or Conference.pt.

83. Conference Paper/ or Conference Paper.pt.

84. Conference Review.pt.

85. Exp "Congresses as Topic"/

86. Consensus Development/

87. Exp Clinical Protocol/ or Clinical Trial Protocol/

88. exp Conference Abstract/ or Conference Abstract.pt.

89. exp Conference Paper/

90. Editorial/ or Editorial.pt.

91. Feasibility Study/

92. Exp Interview/

93. Letter/ or letter.pt.

94. Note/ or Note.pt.

95. Exp Practice Guideline/

© 2026 Beshar I et al. *JAMA Network Open*.

96. Exp Review/ or review.pt.

97. Short Survey/

98. Validation Study/

99. (abstract* or "annual meeting" or book or "case report*" or "case series" or comment* or conference or congress or editor* or feasibility or interview* or letter or "meta-analysis" or meeting or note or paper* or pilot or poster* or proceeding* or protocol or review or symposium or validation).ti.

100. OR/75-99 [irrelevant publication types]

101. 74 NOT 100 [results without irrelevant publication types]

102. Limit 101 to english

103. Limit 102 to yr="1990-current"

OVID MEDLINE

1. "Hereditary Breast and Ovarian Cancer Syndrome"/
2. *BRCA1* protein/
3. *BRCA2* protein/
4. "Genes, *BRCA1*"/
5. "Genes, *BRCA2*"/
6. (*BRCA1* or *BRCA2*).ti,ab.
7. (BRCA*1 or BRCA*2).ti,ab.
8. (hereditary adj3 (breast or ovarian) adj3 cancer*).ti,ab.
9. (familial adj3 (breast or ovarian) adj3 cancer*).ti,ab.

© 2026 Beshar I et al. *JAMA Network Open*.

10. HBOC.ti,ab.
11. (genetic* predispos* adj3 (breast or ovarian) adj3 cancer*).ti,ab.
12. OR/1-11 [HBOC]
13. Exp Prophylactic Surgical Procedure/
14. ((preemptive or "pre-emptive" or prophylactic* or prevent* or "risk-reduc*" or "risk reduc*") ADJ2 (surger* or surgical or procedure*)).ti,ab.
15. Prophylactic Mastectomy/
16. ((bilateral or double or contralateral) adj3 mastectom*).ti,ab.
17. ((preemptive or pre-emptive or prophylactic* or preventiv* or prevent* or risk-reduc* or "risk reduc*") ADJ2 (mastectom*)).ti,ab.
18. Salpingo-Oophorectomy/
19. ("risk-reduc*" ADJ2 "salpingo-oophorectom*").ti,ab.
20. Exp Salpingectomy/
21. Exp Salpingo-oophorectomy/
22. (oophorectom* or ovariectom* or salpingectom* or "salpingo-oophorectom*").ti,ab.
23. OR/13-22 [preventative surgeries]
24. Exp Mammography/
25. (mammograph* or mammogram* or "breast magnetic resonance" or "breast computed tomograph*" or echomammogra* or xeromammography).ti,ab.
26. OR/24-25 [mammograms]
27. Exp "Contraceptives, Oral"/
28. ("oral contraceptive*" or "oral contraception" or "birth control pill*" or "contraceptive agent*" or "contraceptive pill*").ti,ab.

© 2026 Beshar I et al. *JAMA Network Open*.

29. (Chlormadinone or Cyproterone or Deposiston or Desogestrel or Drospirenone or Estradiol or "Ethinyl Estradiol" or Etynodiol or Levonorgestrel or Lynestrenol or Medroxyprogesterone or Mestranol or Norethisterone or Norethindrone or Norgestrel or Meloxifene or Segesterone or Tanaproget).Ti,Ab.
30. Anastrozole/
31. (Anastrozole or Arimidex or Femathina or Zolitrat).ti,ab.
32. Exp Aromatase inhibitor/
33. (aromasin or "aromatase inhibitor*" or aminoglutethimide or exemestane or letrozole).ti,ab.
34. Raloxifene/
35. (Evista or Loxifen or Ortruma or Ostiral or Raloxifene).ti,ab.
36. Selective Estrogen Receptor Modulator /
37. ("estrogen receptor modulat*" or "oestrogen receptor modulat*").ti,ab.
38. Tamoxifen/
39. Tamoxifen Citrate/
40. (Tamoxifen or Nolvadex or Tamofen* or Soltamox).ti,ab.
41. Exp Intrauterine Contraceptive Devices/
42. ("intrauterine contraceptive device*" or "intrauterine device*" or IUD or "I.U.D." or levonorgestrel).ti,ab.
43. OR/27-42 [all drug terms]
44. OR/23,26,43 [prophylactic surgeries, mammograms, contraception]
45. AND/12,44 [HBOC and interventions]
46. Mortality/
47. Survival Rate/

© 2026 Beshar I et al. *JAMA Network Open*.

48. (cancer adj2 (mortalit* or death or fatalit*)).ti,ab.

49. ((death or fatality or mortality) ADJ rate).ti,ab.

50. "all-cause mortalit*".ti,ab.

51. ("overall survival" or "cancer-specific survival").ti,ab.

52. "survival outcome*".ti,ab.

53. (survival or mortality or death*).ti.

54. Ovarian Neoplasms/mo [Mortality]

55. (("ovar* cancer" or "ovar* carcinoma" or "ovar* metasta*") ADJ2 (death* or mortality)).ti,ab.

56. Breast Neoplasms/mo [Mortality]

57. (("breast cancer" or "breast carcinoma" or "breast metasta*") ADJ2 (death* or mortality)).ti,ab.

58. Endometrial Neoplasms/mo [Mortality]

59. (("endometrial cancer" or "endometrial carcinoma" or "endometrial metasta*") ADJ2 (death* or mortality)).ti,ab.

60. Colorectal Neoplasms/mo [Mortality]

61. (("colo* cancer" or "colo* carcinoma" or "colo* metasta*") ADJ2 (death* or mortality)).ti,ab.

62. Fallopian Tube Neoplasms/mo [Mortality]

63. (("fallopian tube* cancer*" or "fallopian tube* carcinoma" or "fallopian tube* metasta*") ADJ2 (death* or mortality)).ti,ab.

64. Proportional Hazards Models/

65. Exp Survival Analysis/

66. "survival analys*".ti,ab.

67. ("relative risk*" or "absolute risk*").ti,ab,kw.

68. (hazard adj2 ratio*).ti,ab,kw.

© 2026 Beshar I et al. *JAMA Network Open*.

69. ("HR" ADJ2 (confidence or CI)).ti,ab,kw.
70. ("cox regression" or "cox model*" or "cox multivariate" or "cox survival").ti,ab,kw.
71. ("proportional hazard*" or "hazard ratio*").ti,ab,kw.
72. OR/46-71 [mortality reduction terms]
73. AND/45,72 [HBOC and interventions and mortality]
74. (exp Animals/ NOT humans.sh.)
75. (amphibian* or animal? or bird or birds or bovine or canine or cat or cats or cattle or cow or cows or dog or dogs or ferret* or fish or frog* or "guinea pig*" or hamster* or horse* or "in vitro" or lambs or llama* or marmoset* or mice or monkey* or mouse or murine or pig or pigs or piglets or porcine or primate or primates or rabbit* or rat or rats or sheep or swine or trout* or veterinar* or zebra*).ti.
76. OR/74-75 [animal studies]
77. 73 NOT 76 [results without animal studies]
78. Case reports/
79. Clinical Trial Protocol/
80. Comment/
81. Exp Congress/
82. Exp "Congresses as Topic"/
83. Editorial/
84. English Abstract/
85. Exp Historical Article/
86. Letter/
87. Overall/

© 2026 Beshar I et al. *JAMA Network Open*.

88. Exp Review/
89. Validation Study/
90. Video-Audio Media/
91. (abstract* or "annual meeting" or "case report" or "case series" or comment* or conference or congress or editor* or feasibility or "in vitro" or letter or "meta-analysis" or meeting or paper* or pilot or poster* or proceeding* or protocol or review or symposium or validation).ti.
92. OR/78-91 [irrelevant publication types]
93. 77 NOT 92 [results without irrelevant publication types]
94. Limit 93 to english
95. Limit 94 to yr="1990-current"

WEB OF SCIENCE

TITLE:

BRCA*1 or BRCA*2 or HBOC or "hereditary breast and ovarian cancer" or HNPCC or "Hereditary Nonpolyposis Colorectal Cancer" or Lynch Syndrome or "familial colorectal cancer" or "hereditary colorectal cancer"

TOPIC:

survival or mortality or death* or "hazard ratio*" or "relative risk reduction*" or "absolute risk*"

TITLE:

© 2026 Beshar I et al. *JAMA Network Open*.

(prophylactic* or prevent* or risk-reduc* or "risk reduc*")

Limited to

English

Articles

© 2026 Beshar I et al. *JAMA Network Open*.

eReferences

1. Wigle A, Beliveau A, Salanti G, et al. Precision of Treatment Hierarchy: A Metric for Quantifying Certainty in Treatment Hierarchies from Network Meta-Analysis.

2. MARIBS Group, Gareth ED, Nisha K, et al. MRI breast screening in high-risk women: cancer detection and survival analysis. *Breast Cancer Res Treat*. 2014;145(3):663-672. doi:10.1007/s10549-014-2931-9

3. Heemskerk-Gerritsen BAM, Menke-Pluijmers MBE, Jager A, et al. Substantial breast cancer risk reduction and potential survival benefit after bilateral mastectomy when compared with surveillance in healthy *BRCA1* and *BRCA2* mutation carriers: a prospective analysis. *Annals of Oncology*. 2013;24(8):2029-2035. doi:10.1093/annonc/mdt134

4. Valero MG, Moo TA, Muhsen S, et al. Use of bilateral prophylactic nipple-sparing mastectomy in patients with high risk of breast cancer. *British Journal of Surgery*. 2020;107(10):1307-1312. doi:10.1002/bjs.11616

5. Warner E, Zhu S, Plewes DB, et al. Breast Cancer Mortality among Women with a *BRCA1* or *BRCA2* Mutation in a Magnetic Resonance Imaging Plus Mammography Screening Program. *Cancers*. 2020;12(11):3479. doi:10.3390/cancers12113479

6. Domchek SM, Friebel TM, Singer CF, et al. Association of Risk-Reducing Surgery in *BRCA1* or *BRCA2* Mutation Carriers With Cancer Risk and Mortality.

7. Passaperuma K, Warner E, Causer PA, et al. Long-term results of screening with magnetic resonance imaging in women with BRCA mutations. *Br J Cancer*. 2012;107(1):24-30. doi:10.1038/bjc.2012.204

8. Evans DGR, Clayton R, Donnai P, Shenton A, Lalloo F. Risk-reducing surgery for ovarian cancer: outcomes in 300 surgeries suggest a low peritoneal primary risk. *Eur J Hum Genet*. 2009;17(11):1381-1385. doi:10.1038/ejhg.2009.60

9. Willert CB, Mellemkjær L, Tolver A, et al. Time trends, uptake, and oncological effects of risk-reducing surgeries in 3067 Danish *BRCA1*/2 carriers: a population-based study with matched controls. *Breast Cancer Res Treat*. 2025;214(3):355-366. doi:10.1007/s10549-025-07821-4

10. Gandhi A, Howell SJ, Harkness EF, et al. Survival Outcomes With or Without Risk-Reducing Mastectomy in *BRCA1* and *BRCA2* Pathogenic Variant Carriers. *J Clin Oncol*. Published online February 4, 2026:JCO-25-00834. doi:10.1200/JCO-25-00834

11. Ingham SL, Sperrin M, Baildam A, et al. Risk-reducing surgery increases survival in *BRCA1*/2 mutation carriers unaffected at time of family referral. *Breast Cancer Res Treat*. 2013;142(3):611-618. doi:10.1007/s10549-013-2765-x

© 2026 Beshar I et al. *JAMA Network Open*.

12. Heemskerk-Gerritsen BAM, Jager A, Koppert LB, et al. Survival after bilateral risk-reducing mastectomy in healthy *BRCA1* and *BRCA2* mutation carriers. *Breast Cancer Res Treat*. 2019;177(3):723-733. doi:10.1007/s10549-019-05345-2

13. Metcalfe K, Huzarski T, Gronwald J, et al. Risk-reducing mastectomy and breast cancer mortality in women with a *BRCA1* or *BRCA2* pathogenic variant: an international analysis. *Br J Cancer*. 2024;130(2):269-274. doi:10.1038/s41416-023-02503-8

14. Record SM, Thomas SM, Ntowe K, Chiba A, Plichta JK. *BRCA1*/2 mutation carriers & risk reducing mastectomy: Who undergoes surgery and potential benefits. *The American Journal of Surgery*. 2024;227:146-152. doi:10.1016/j.amjsurg.2023.10.011

15. Lubinski J, Kotsopoulos J, Moller P, et al. MRI Surveillance and Breast Cancer Mortality in Women With *BRCA1* and *BRCA2* Sequence Variations. *JAMA Oncol*. 2024;10(4):493. doi:10.1001/jamaoncol.2023.6944

© 2026 Beshar I et al. *JAMA Network Open*.
