# Supplementary material for: Survival After Risk-Reducing Mastectomy in Patients With Pathogenic Variants in BRCA1 and BRCA2: A Network Meta-Analysis
Source: JAMA Netw Open. 2026 Oct 30;9(10):e2641231. doi: 10.1001/jamanetworkopen.2026.41231 (PMC13723018; doi:10.1001/jamanetworkopen.2026.41231)
Supplement: Supplementary file 2 [file jamanetwopen-e2641231-s002.pdf]

# Data Sharing Statement

Beshar. Survival After Risk-Reducing Mastectomy in Patients With Pathogenic Variants in BRCA1 and BRCA2. *JAMA Netw Open*. Published October 30, 2026.
doi:10.1001/jamanetworkopen.2026.41231

**Data**
**Data available:** Yes
**Data types:** Data (not involving human participants)
**How to access data:** Data will be made available upon request. Data includes summary-level data from included studies and detailed statistical analysis plan. Data can be made available at time of publication.
**When available:** With publication

**Supporting Documents**
**Document types:** None

**Additional Information**
**Who can access the data:** Upon reasonable request
**Types of analyses:** For purposes of additional research
**Mechanisms of data availability:** With investigator support
